# Effect of hypofractionation on the incidental axilla dose during tangential field radiotherapy in breast cancer

**DOI:** 10.1007/s00066-020-01636-6

**Published:** 2020-06-02

**Authors:** Kai J. Borm, Markus Oechsner, Mathias Düsberg, Gabriel Buschner, Weber Wolfgang, Stephanie E. Combs, Marciana N. Duma

**Affiliations:** 1 Department of RadiationOncology, Technical University Munich, Medical School, Klinikum rechts der Isar, Munich, Germany; 2 Department of Nuclear Medicine, Technical University Munich, Medical School, Klinikum rechts der Isar, Munich, Germany; 3 Deutsches Konsortium für Translationale Krebsforschung (DKTK)-Partner Site Munich, Munich, Germany; 4 Institute of Radiation Medicine, Helmholtzzentrum München, Ingolstaedter Landstr. 1, 85764 Neuherberg, Germany; 5 Department of Radiotherapy and Radiation Oncology, Friedrich Schiller University Hospital, Bachstraße 18, 07743 Jena, Germany

**Keywords:** Biological effective dose, Tumor control probability, Incidental axillary dose, Sentinel node

## Abstract

**Objective:**

Tangential field irradiation in breast cancer potentially treats residual tumor cells in the axilla after sentinel lymph node biopsy (SLNB). In recent years, hypofractionated radiotherapy has gained importance and currently represents the recommended standard in adjuvant breast cancer treatment for many patients. So far, the impact of hypofractionation on the effect of incidental lymph node irradiation has not be addressed.

**Materials and methods:**

Biological effective dose (BED) and tumor control probability (TCP) were estimated for four different hypofractionated radiation schemes (42.50 Gy in 16 fractions [Fx]; 40.05 Gy in 15 Fx; 27 Gy in 5 Fx; and 26 in 5 Fx) and compared to conventional fractionation (50 Gy in 25 Fx). For calculation of BED and TCP, a previously published radiobiological model with an α/β ratio of 4 Gy was used. The theoretical BED and TCP for incidental irradiation between 0 and 100% of the prescribed dose were evaluated. Subsequently, we assessed BED and TCP in 431 axillary lymph node metastases.

**Results:**

The extent of incidental lymph node irradiation and the fractionation scheme have a direct impact on BED and TCP. The estimated mean TCP in the axillary nodes ranged from 1.5 ± 6.4% to 57.5 ± 22.9%, depending on the patient’s anatomy and the fractionation scheme. Hypofractionation led to a significant reduction of mean TCP of lymph node metastases for all schedules.

**Conclusion:**

Our data indicate that hypofractionation might affect the effectiveness of incidental radiotherapy in the axilla. This is particularly relevant for patients with positive sentinel lymph nodes who receive SLNB only.

## Introduction

The Z0011 trial [1] demonstrated that use of sentinel lymph node biopsy (SLNB) alone compared with axillary lymph node dissection (ALND) does not result in inferior survival in patients with one or two positive sentinel lymph nodes. Interestingly, the regional recurrence rate after SLNB alone was very low (<1%) even though approximately 27% of patients had lymph node metastases in the undissected axillary nodes. Since tangential field irradiation delivers a relevant dose to the axillary levels, adjuvant radiotherapy contributes to eradication of microscopic disease in the undissected axilla and accounts for the good oncologic outcomes after SLNB only. In accordance with this, current guidelines recommend omitting ALND only if patients receive systemic therapy and adjuvant radiotherapy (RT) [1, 2]. The MA-20 [3] and EORTC 22922-10925 [4] studies have shown that elective lymph node irradiation (supra/infraclavicular and internal mammary) is effective in lowering regional and distant metastases and prolongs disease-free survival, which emphasizes the potential of lymph node irradiation in breast cancer.

Several previous studies focused on the dose distribution in the axillary levels during tangential field irradiation [5–8].The dose distribution varies largely depending on patient anatomy and treatment technique (e.g., high tangent vs. conventional tangent). Usually, a smaller dose per fraction compared to the prescribed dose is delivered to the axillary lymph node areas [9]. This has a direct impact on the biological effective dose (BED) and tumor control probability (TCP) of microscopic disease in the axillary lymph nodes [9–11].

Nowadays, moderate hypofractionated radiotherapy (40–42.5 Gy in 15–16 fractions [Fx]) is the recommended therapy for adjuvant radiotherapy after breast-conserving surgery for most patients [1, 12–14]. Several trials have been initiated to test the outcome of even higher doses per day (extreme hypofractionation). The UK FAST trial [14] tested 30 or 28.5 Gy in 5 Fx once a week and showed promising results with only two local relapses in 915 patients at 3 year median follow up. The UK FAST-Forward trial (*N* = 4000) [15] recruited patients between 2012 and 2014 and tested 27 or 26 Gy in 5 Fx/week against 40 Gy in 15 Fx. Since the study remains in follow-up, results are pending.

Nonetheless, two different approaches have changed simultaneously in breast cancer treatment: hypofractionation is considered a standard therapy and at the same time, de-escalation of axillar surgery is conducted for an increasing number of patients. The effect of fractionation on the incidental radiotherapy dose to the axilla during whole-breast radiotherapy has not been addressed in the literature so far. Thus, it remains unclear whether hypofractionated radiotherapy might impair the local outcome in the axilla.

The aim of the current study was to evaluate if different fractionation schedules have a potential effect on TCP during incidental lymph node irradiation in the areas at risk.

## Materials and methods

In a previous study, 580 F18-FDG-PET/CT-positive lymph node metastases of breast cancer patients were detected and mapped in a CT template [16]. 431 of these 580 lymph nodes were located in axillary levels I–III. Using the previously published methodology, these 431 lymph node metastases were registered rigidly and non-rigidly to the three different patients listed in Table 1. The patients were chosen to represent different breast sizes (ranging from small to large) and breast shape. The dose in every lymph node was assessed in each patient for the equilateral tangent treatment plan and the different prescription doses. The dosimetric data was then transferred to SPSS (IBM statistics version 25, IBM Corp., Armonk, NY, USA).

**Table 1:** Patient characteristics

Patient	Age (years)	BMI	Bust girth^a^	Breast Volume
1	50	26.6 kg/m^2^	98 cm	763 cm^3^
2	53	26.0 kg/m^2^	107 cm	1201 cm^3^
3	48	20.6 kg/m^2^	85 cm	329 cm^3^

^a^taken at the nipple line

### Treatment planning

Planning CTs of the three patients were acquired on a Somatom Emotion scanner (Siemens medical solutions, Erlangen, Germany). The planning kilovoltage computed tomography (CT) scan was performed in free breathing (FB) with both arms over the head. The slice thickness of the CTs was 3 mm and no contrast agent was applied. Contouring and treatment planning were performed with the Eclipse 13.0 Treatment Planning System (Varian Medical Systems, Palo Alto, CA, USA). Contouring of the planning target volume (PTV) and organs at risk (OAR) was performed according to the RTOG breast-contouring atlas [17] in all CT scans. The clinical target volume (CTV) to PTV margin for the breast was 1 cm, with inclusion of the chest wall in the PTV. A PTV was generated for the right and for the left side, and for each side a treatment plan was calculated. The treatment plan consisted of two opposing tangential beams with an additional 1–5 beam segments to improve target dose coverage and homogeneity. The dose was prescribed to the median dose in the PTV. The prescribed fractionation schedules are summarized in Table 2 including total dose (D), dose per fraction (d), and number of fractions (Fx). The prescribed dose was modified according to Table 2 for the tangential treatment plans without any changes of the field design.

**Table 2:** Different fractionation schemes used in adjuvant radiotherapy (RT) of the breast

Schedule	Total dose (D)	Dose per fraction (d)	Number of fractions
Conventional RT	50 Gy	2.00 Gy	25
Hypofractionated RT 1 [18]	42.5 Gy	2.66 Gy	16
Hypofractionated RT 2 [19]	40 Gy	2.67 Gy	15
UK FAST-Forward 1 [15]	27 Gy	5.40 Gy	5
UK FAST-Forward 2 [15]	26 Gy	5.20 Gy	5

### BED and TCP

In 2009, Planatiotis and Dale [11] published a study in the *International Journal of Radiation Oncology, Biology, Physics* linking BED to TCP based on the existing randomized trials of RT vs. non-RT in breast cancer including hypofractionated RT schedules.

For calculation of BED and TCP, Excel (Microsoft Corporation, version 16, Redmond, Washington, United States) and SPSS (IBM statistics) were used. The biological effective dose was calculated according to Eq. 1.

1 $$\mathrm{BED}=\mathrm{Fx*}\mathrm{d} \left( 1+\frac{\mathrm{d}}{\upalpha /\upbeta }\right)$$

Correspondingly to the study of Planatiotis and Dale [11], we used an α/β value of 4 Gy for breast cancer cells. The BED for every lymph node (representing areas at risk of containing microscopic disease) in the three patients was calculated in dependence of the fractionation schedules (Table 2). Thus, a total of 12,900 dose values were evaluated. An example can be found in Eq. 2 for a lymph node (LN1) receiving 50% (1 Gy) of the prescribed dose (2 Gy) for 25 fractions.

2 $$\mathrm{BED}_{\mathrm{LN}1 }=25\mathrm{*} 1\,\mathrm{Gy} \left(1+\frac{1\,\mathrm{Gy}}{4\,\mathrm{Gy}}\right)=31.2\,\mathrm{Gy}$$

Furthermore, a diagram was calculated linking the extent of incidental irradiation (x-axis: 0–100% of the prescribed dose) to the BED (y-axis) for different fractionation schemes.

3 $$\mathrm{f}\left(\mathrm{x}\right)=\mathrm{Fx*} \frac{\mathrm{x}}{100}\times \mathrm{*} \left(1+\frac{\mathrm{d}\times \mathrm{x}}{400\,\mathrm{Gy}}\right)$$

For calculation of the TCP the equation, Eq. 4, given in the publication by Planatiotis and Dale was used. TCP was defined as:

$$\mathrm{TCP}=\frac{\text{failure rate without RT}-\text{failure rate with RT}}{\text{failure rate without RT}}$$

The equation is based on nine studies with a calculated BED ranging from 75 to 96 Gy and TCP ranging from 55.5 to 96.6%:

4 $$\mathrm{TCP}=e^{-59.2\mathrm{*} {e^{-0.07\text{* BED}}}}$$

To estimate the control probability in the areas at risk of containing microscopic disease, TCP in the lymph node metastases was calculated. The dose in the lymph nodes was transferred to SPSS for statistical analysis. Differences between the fractionation schemes were tested for statistical significance using a two-sided paired *t*-test. A *p*-value <0.001 was defined as statistically significant.

## Results

### Estimation of BED and TCP during incidental lymph node irradiation

**Fig. 1:**
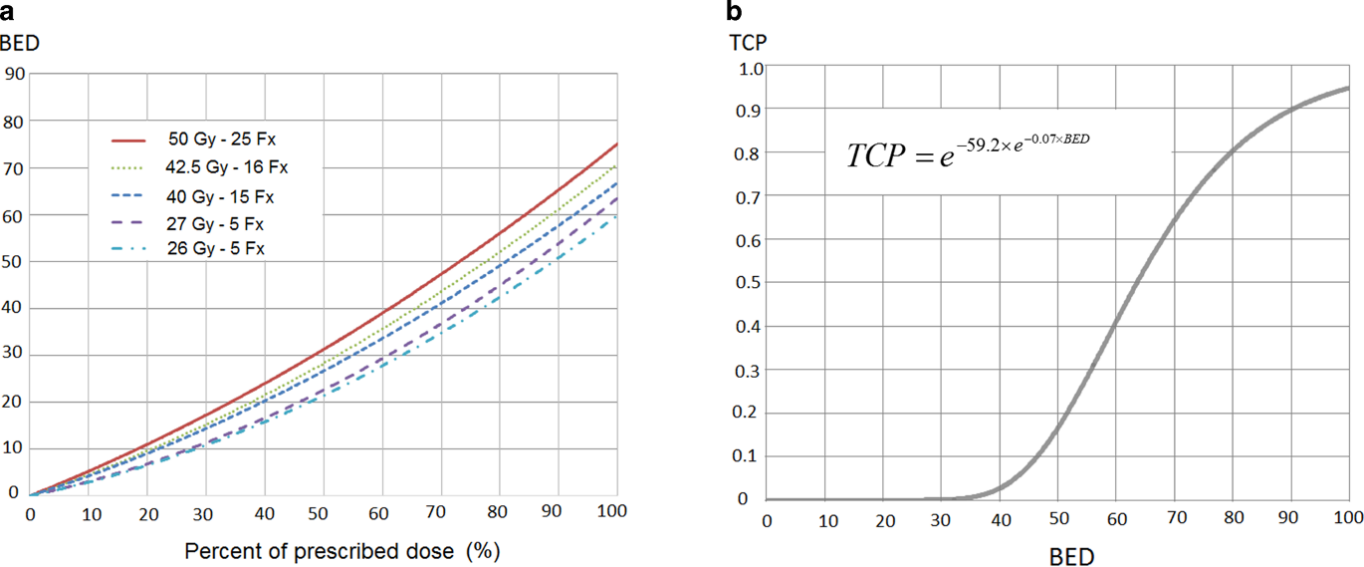
The impact of different fractionation schedules on the effect of incidental lymph node irradiation. **a** Biological effective dose (*BED*) in dependence of the extent of incidental lymph node irradiation (0–100%) compared to the prescribed dose; **b** function and curve linking BED to tumor control probability

Both the extent of incidental irradiation and the fractionation schedules had an important impact on the BED. The BED curves for the different fractionation schedules are presented in Fig. 1. The highest BED values (BED_max_ = 75.0 Gy) were measured for the conventional fractionation schedule and the lowest (BED_max_ = 59.8 Gy) for the FAST-Forward schedule. For moderate hypofractionation with 15 or 16 Fx, the maximal BED difference compared to the conventional fractionation schedule was −8.3 Gy and −4.3 Gy, respectively. The different BEDs resulted in a maximal reduction (absolute) of TCP between −10.4% (42.5 Gy/16 Fx) and −36.0% (26 Gy/5 Fx; Fig. 1c). For a BED <36.4 Gy or incidental irradiation lower than 57% (50 Gy in 25 Fx) to 72% (26 Gy in 5 Fx) of the prescribed dose, no relevant benefit (TCP <1%) could be expected after RT according to the model.

### BED and TCP in axillary lymph node metastases

The treatment plans of the three patients resulted in different extents of incidental dose distribution in the lymph nodes. The average percentage of incidental irradiation in the lymph nodes can be found in Table 3. Patient I and II with larger breast volumes/PTVs showed better coverage of the lymph nodes compared to patient III with a small breast. The best dose coverage was found for level I, with values ranging from 22.2 ± 28.6% to 81.6 ± 28.2%. The lowest dose coverage was observed in level III, with values between 5.4 ± 12.7% and 64.2 ± 36.1%.

**Table 3:** Percent of prescribed dose in the lymph nodes located in the axillary lymph node levels I–III. Mean values ± standard deviation of the three patients

		Patient 1	Patient 2	Patient 3
Level I	*N* = 316	81.6 ± 28.2%	92.3 ± 16.8%	22.2 ± 28.6%
Level II	*N* = 57	79.0 ± 27.8%	90.3 ± 16.3%	13.6 ± 24.1%
Level III	*N* = 58	44.5 ± 39.1%	64.2 ± 36.1%	5.4 ± 12.7%

**Fig. 2:**
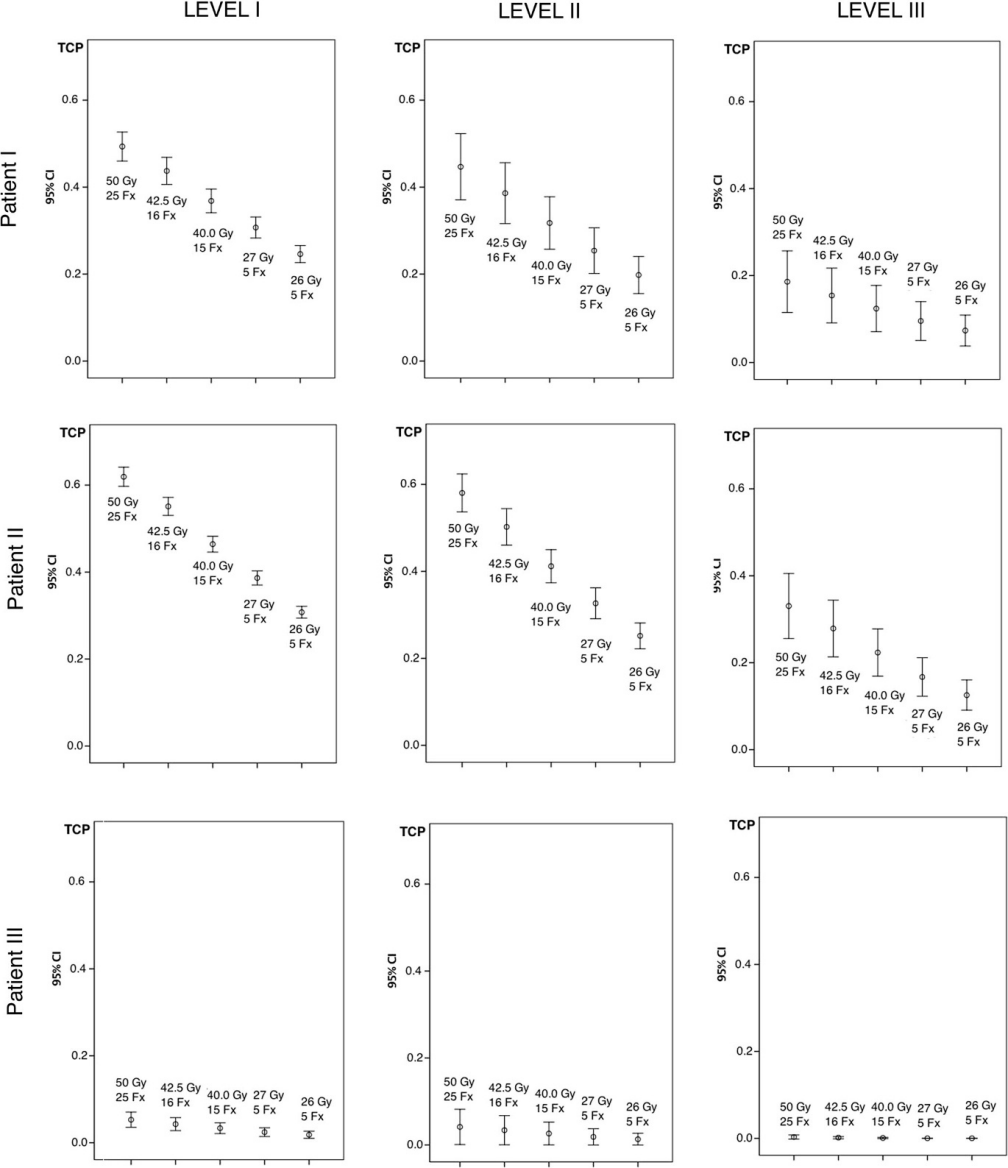
Tumor control probability of 431 lymph nodes receiving incidental lymph node irradiation in three different patients for conventional and hypofractionated radiation schemes. Axillary levels I–III. Patients I–III. Mean values and 95% confidence interval (*CI*). Schedules described as total dose with number of fractions (*Fx*)

The mean BED in the axillary lymph nodes was significantly (*p* < 0.001) lower for all hypofractionated schedules compared to conventional fractionation (Table 4). The largest differences between the fractionation schemes were found in patient II. The mean TCP in the lymph nodes was significantly lower for the hypofractionation schedules according to the model. This was the case for all patients. For both BED and TCP, large standard deviations (SD) of the mean values were observed due to the variable localization of the lymph nodes with respect to the radiation field. Fig. 2 depicts the mean TCP for the axillary levels I–III. As incidental lymph node irradiation decreased from level I to level III, the lowest TCPs were found for lymph nodes located in level III.

**Table 4:** Mean biological effective dose (BED) and tumor control probability (TCP) of the lymph nodes in the three patients with different fractionation schedules. Mean values ± standard deviation

	Patient I		Patient II		Patient III	
Schedule	BED (Gy)	TCP (%)	BED (Gy)	TCP (%)	BED (Gy)	TCP (%)
50 Gy/25 Fx	55.3 ± 25.2	44.6 ± 31.4	64.8 ± 17.8	57.5 ± 22.9	12.1 ± 19.2	4.4 ± 14.4
42.5 Gy/16 Fx	51.8 ± 24.0*	38.5 ± 28.5*	60.8 ± 17.0*	49.9 ± 21.0*	11.0 ± 17.9*	3.5 ± 12.1*
40 Gy/15 Fx	48.9 ± 22.6*	32.9 ± 25.1*	57.5 ± 16.1*	42.5 ± 18.6*	10.4 ± 16.9*	2.8 ± 10.1*
27 Gy/5 Fx	45.6 ± 22.0*	27.2 ± 22.0*	54.0 ± 15.7*	34.9 ± 16.6*	9.0 ± 15.6*	2.0 ± 8.1*
26 Gy/5 Fx	43.1 ± 20.7*	21.7 ± 18.0*	50.9 ± 14.8*	27.6 ± 13.7*	8.5 ± 14.7*	1.5 ± 6.4*

*Fx* fraction, *BED* biological effective dose, *TCP* tumor control probability, *Gy* Gray

*significant difference compared to the standard fractionation schedule (*p* < 0.001)

## Discussion

Current guidelines recommend the omission of ALND in case of 1) negative SLNB, 2) micrometastases seen in SLN, or 3) 1–2 positive lymph nodes and T1–2 tumors after BCS without preoperative chemotherapy and planned whole-breast RT [5]. According to these recommendations, residual tumor cells in the axillary lymph nodes must be expected in a relevant number of patients prior to whole-breast irradiation. Large randomized trials (START Pilot, START A/B, Ontario trial) suggest that hypofractionated radiotherapy can be safely used in most breast cancer patients even if the total dose is reduced by approximately 10%. In 2018 an updated American Society for Radiation Oncology (ASTRO) evidence-based guideline was published, recommending hypofractionation as the standard scheme during whole breast irradiation [1]. However, the effect of incidental irradiation in the axilla remains unclear in these studies. The START trials [20] reported only relapses in the ipsilateral axilla if it had been within the irradiated target volumes. Regional relapses outside the radiotherapy target volume were excluded from the analysis of locoregional relapse. In the Ontario trial [21], all patients were treated with axillary dissection and were staged as pN0. Furthermore, axillary relapses were not reported separately and can therefore not be compared between the two schedules. Furthermore, in the Z0011 trial, which is one of the foundations of today’s guidelines, high tangents were used in approximately 50% of the patients and 17% received supraclavicular fields [17]. Hence, the outcome of patients treated with SLNB only receiving hypofractionated radiotherapy to the breast remains unclear.

The TCPs in our study were calculated according to a model by Plataniotis and Dale [10]. The authors defined TCP as *(failure rate without RT − failure rate with RT)/failure rate without RT.* Thus, the calculated values relate only to the beneficial effect of radiotherapy on local control. When interpreting our data, it needs to be taken into consideration that TCPs most likely do not correctly reflect the actual expected tumor control in axillary lymph nodes. Firstly, because the model was calculated for local breast cancer recurrences not for axillary recurrences. A pre-RT average clonogen number per tumor of 59.2 was assumed, which does not necessarily reflect the clonogen in the axillary lymph nodes in case of residual tumor. Secondly, the datapoints used for the model are all clustered around relatively high BEDs, causing considerable uncertainties for low BEDs. In addition, Plataniotis and Dale et al. used an alpha/beta ratio of 4 for calculation of BEDs. However, the alpha/beta ratio of breast cancer cells is still controversial and recent analyzes proposed values clearly lower than 4 [22]. The alpha/beta ratio has a direct impact on the calculated BEDs as well as the TCP formula. Despite these limitations, the publication currently provides the best model linking BED and TCP for microscopic breast cancer cells taking both hypofractionated and normofractioned schemes into account. Even though the model might not predict the exact control probability in the lymph nodes, it clearly helps to understand the effect of hypofractionation during incidental irradiation.

In accordance with other studies, the model by Plataniotis and Dale suggest an s‑shape of the TCP curve, with increasing slope from low to moderate BEDs and increasing slope from moderate to high BEDs [10, 20]. Thus, for high BEDs (e.g., in the boost target volume), changes of BED result in minor changes of TCP, whereas changes of lower BEDs during incidental irradiation result in large differences of TCP due to the steepness of the slope (Fig. 1). Our study focuses on the effect of hypofractionation on local control, even though the rate of axillary recurrences is very low in breast cancer patients. Nevertheless, taking the results from the MA-20 [3] and EORTC studies [4] into account, treatment of microscopic disease in the lymph node system has a potential effect on distant metastases and disease-free survival. Thus, hypothetically, the TCP in the axillary lymph node regions might not only affect locoregional recurrences. However, patients without an indication for regional lymph node irradiation usually have a lower baseline risk of lymphatic tumor spread. Thus, it is unclear whether the (theoretical) reduction of TCP has a (measurable) clinical impact in early breast cancer patients and hypofractionation remains the standard regimen for most patients.

As tangential field irradiation is the standard technique for breast radiotherapy in many departments [21, 23], we analyzed the dose distribution to the axillary lymph nodes during tangential field irradiation. Several studies report average doses in the axillary lymph node levels that range widely in dependence of patient anatomy [6, 9, 11]. To account for differences related to anatomy and thus field design, we choose three different patients with very different breast sizes and shapes. Hence the degree of incidental lymph node irradiation varied between the patients from 12.1 ± 19.2% of the prescribed dose (patient III) to 64.8 ± 17.8% of the prescribed dose (patient II). Differently from our study, most previous studies estimated the dose distribution in the lymph node areas as defined by RTOG or ESTRO guidelines [5, 6, 9, 11, 24, 25]. However, these guidelines define a treatment volume and do not necessary reflect the whole axillary lymph node drainage system and both primary lymph node metastases and lymph node recurrences occur outside the ESTRO and RTOG margins [16, 26]. The 2018 published 3D lymph node atlas [16] allows dose evaluation and calculation of the TCP for actual “areas at risk” in which lymph node metastases frequently occur. Still, it should be considered that detected areas at risk in the lymph node atlas (including also recurrent and metastatic breast cancer patients) might not be representative for primary early breast cancer patients receiving SLNB only.

The total treatment time between conventional fractionation and the analyzed hypofractionated schemes differed by up to 28 days. Based on the START trials, previous studies generated the hypothesis that overall treatment time is a significant determinant of local cancer control. The assumption was prompted by a suggestion of lower locoregional relapse rates after 40 Gy in 15 fractions in in START‑B trial. Based on the START trials, Haviland et al. estimated an overall treatment time effect for locoregional relapse of 0.6 Gy/day [27]. However, the presumed time effect conflicts with the results of the Ontario trial testing 42.5 Gy in 16 fractions without any differences in long-term outcome. Furthermore, the literature regarding the impact of a treatment delay are inconsistent and the repopulation kinetics of microscopic breast cancer are known to be slow [28]. In the model by Plataniotis and Dale [10], repopulation during treatment was assumed to be small and was not taken into account. Even though the subject remains controversial, neglecting the potential impact of overall treatment time in the TCP formula (and therefore in our calculations) can be seen as a relevant limitation of our study. Given the limitations of the TCP calculation, the level of significance was set at <0.001 to keep statistical uncertainties as low as possible.

For a better accuracy of the model, we analyzed only schemes with 5 Fx per week. In addition to the recommended (standard) fractionation of 42.67 or 40.05 Gy, we analyzed the FAST-Forward schemes. These schedules are yet not recommended, but currently being tested in prospective trials [15]. Since the UK-FAST trial showed promising results after 3 years of follow-up, extreme hypofractionation with higher single doses could become more important. Thus, the potential effect of hypofractionation in the axillary lymph nodes should be considered for further prospective studies as well as during interpretation of results.

## Conclusion

According to the available radiobiological models, hypofractionated RT leads to lower TCPs in areas of risk containing microscopic disease in the axilla compared to normofractioned RT. Due to low rates of axillary recurrences in early breast cancer, it is unclear whether this finding has a clinical significance. Nevertheless, special attention should be paid to this issue in further studies on this subject.
